# Simian Varicella Virus Infection of Rhesus Macaques Recapitulates Essential Features of Varicella Zoster Virus Infection in Humans

**DOI:** 10.1371/journal.ppat.1000657

**Published:** 2009-11-13

**Authors:** Ilhem Messaoudi, Alexander Barron, Mary Wellish, Flora Engelmann, Alfred Legasse, Shannon Planer, Don Gilden, Janko Nikolich-Zugich, Ravi Mahalingam

**Affiliations:** 1 Vaccine and Gene Therapy, Division of Pathobiology and Immunology, Oregon National Primate Research Center, Oregon Health and Sciences University, Beaverton, Oregon, United States of America; 2 Department of Neurology, University of Colorado Denver, Aurora, Colorado, United States of America; 3 Department of Microbiology, University of Colorado Denver, Aurora, Colorado, United States of America; 4 Department of Immunobiology, University of Arizona, Tucson, Arizona, United States of America; National Institutes of Health, United States of America

## Abstract

Simian varicella virus (SVV), the etiologic agent of naturally occurring varicella in primates, is genetically and antigenically closely related to human varicella zoster virus (VZV). Early attempts to develop a model of VZV pathogenesis and latency in nonhuman primates (NHP) resulted in persistent infection. More recent models successfully produced latency; however, only a minority of monkeys became viremic and seroconverted. Thus, previous NHP models were not ideally suited to analyze the immune response to SVV during acute infection and the transition to latency. Here, we show for the first time that intrabronchial inoculation of rhesus macaques with SVV closely mimics naturally occurring varicella (chickenpox) in humans. Infected monkeys developed varicella and viremia that resolved 21 days after infection. Months later, viral DNA was detected only in ganglia and not in non-ganglionic tissues. Like VZV latency in human ganglia, transcripts corresponding to SVV ORFs 21, 62, 63 and 66, but not ORF 40, were detected by RT-PCR. In addition, as described for VZV, SVV ORF 63 protein was detected in the cytoplasm of neurons in latently infected monkey ganglia by immunohistochemistry. We also present the first in depth analysis of the immune response to SVV. Infected animals produced a strong humoral and cell-mediated immune response to SVV, as assessed by immunohistology, serology and flow cytometry. Intrabronchial inoculation of rhesus macaques with SVV provides a novel model to analyze viral and immunological mechanisms of VZV latency and reactivation.

## Author Summary

Varicella zoster virus (VZV) is a ubiquitous herpesvirus that causes chickenpox (varicella) and establishes a life-long latent infection in humans. VZV can then reactivate causing herpes zoster, commonly known as shingles, a painful and debilitating disease that can be life-threatening in elderly and immunocompromised individuals. Our understanding of immunological factors that control VZV replication is limited due to the fact that VZV causes disease only in humans. Simian varicella virus (SVV) is a simian counterpart of VZV that infects nonhuman primates. Our study showed for the first time that infection of rhesus macaques with SVV recapitulates key aspects of VZV infection from chickenpox and the resolution of acute viremia to the establishment of latency in ganglia, and the development of B and T cell responses to virus. This model will allow us to improve our understanding of the immune response to varicella virus, and to develop and test vaccines and antiviral drugs that can reduce the incidence of shingles and its attendant neurological complications.

## Introduction

Varicella zoster virus (VZV), a neurotropic alpha herpesvirus, is the etiological agent of varicella (chickenpox). VZV establishes latency in ganglia and can reactivate to produce herpes zoster (shingles), a debilitating disease for the elderly and immunocompromised. Studies of VZV pathogenesis have been hampered by the lack of an animal model that consistently recapitulates both the virological and immunological hallmarks of both acute and latent VZV infection. Experimental inoculation of mice, rats and nonhuman primates (NHP) with VZV results in seroconversion but not varicella [1],[2],[3]. In weanling guinea pigs, seroconversion, viremia, an exanthem and animal-to-animal spread after VZV infection have been found [4]. Although humoral and cellular immune responses have been briefly characterized in the guinea pig model, the scarcity of immunological tools specific for this species have precluded in depth analysis [5],[6]. Further, VZV does not reactivate in any of these models. A SCID-humanized (SCID-hu) mouse model was developed in which co-implants of human fetal thymus/liver tissue were introduced under the kidney capsule. Fetal skin was then introduced subcutaneously as full thickness dermal grafts and later infected by injection of VZV-infected cells into the implanted tissue [7]. Further studies showed that the skin implants can also be infected and display hallmarks of VZV lesions following intravenous transfer of VZV infected tonsilar T cells [8]. Although this model contributed significantly to our understanding of VZV dissemination and pathogenesis, the chimeric and partially immunodeficient status of the host coupled with the need for fetal liver, thymus, tonsils and skin, precluded in depth studies of the role of the host immune response in the establishment and maintenance of VZV latency.

Simian varicella virus (SVV) produces a naturally occurring exanthematous disease in NHP that mimics human varicella [9]. Both virus genomes have been sequenced and are colinear, sharing up to 75% DNA similarity [10],[11],[12]. Furthermore, SVV and VZV encode antigenically related polypeptides [13],[14],[15]. Like VZV, SVV becomes latent in ganglionic neurons [16],[17],[18],[19] and reactivates after environmental stress or immune suppression [9],[20],[21],[22]. However, although intratracheal inoculation of African green or Cynomolgus monkeys with SVV produces clinical and pathological features in monkeys similar to those seen in humans infected with VZV [9],[23], the animals remain persistently viremic for years [24],[25]. Thus, it is not ideal to use these models to characterize immunological control of VZV infection.

To address this limitation and study VZV latency, a model of natural SVV infection was developed whereby African Green or Cynomolgus monkeys were exposed to cage-mates that had been experimentally inoculated with SVV [21],[26]. While natural infection resulted in rash and SVV DNA was detected only in ganglia and not in liver or lung, features consistent with varicella latency, seroconversion was random, and viremia was not found in most animals. These observations, coupled with an unknown dose and exact time of infection, make it difficult to study the kinetics of the host immunological response to virus infection [21]. The goal of this study was to investigate whether intrabronchial inoculation of rhesus macaques with SVV would provide a more suitable model of VZV pathogenesis and latency. In this manuscript, we report that intrabronchial infection of rhesus macaques with SVV reproduced all of the cardinal features of primary VZV infection in humans.

## Results

### Intrabronchial inoculation of rhesus macaques with SVV results in varicella, viremia and viral latency

**Figure 1:**
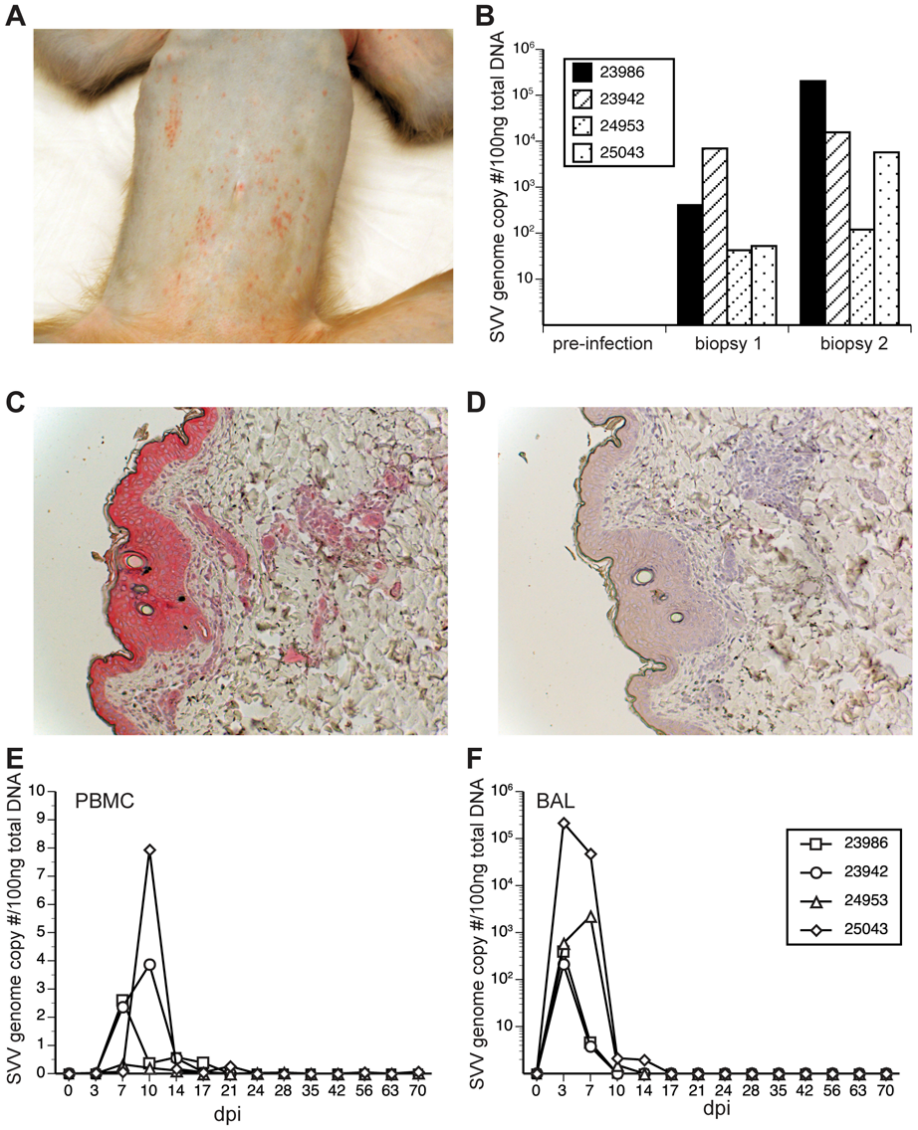
Varicella and the presence of SVV DNA and viral antigen in rhesus macaques inoculated intrabronchially with SVV. (A) Varicella rash on skin of monkey 24953 9 days after intrabronchial infection. (B) Detection of SVV DNA by real-time PCR in punch biopsies of skin from all 4 monkeys during varicella. (C) Immunohistochemical detection of SVV antigen with antibodies directed against SVV glycoproteins H and L in skin of monkey 24953 during varicella, with staining in superficial and deep layers of skin. (D) Skin staining of monkey 24953 with normal rabbit serum. (E) SVV DNA levels in peripheral blood mononuclear cells (PBMC) and samples of all monkeys as assessed by quantitative real-time PCR. SVV DNA was first detected 7 days post-infection (dpi), peaked at 10 dpi and was not detected 21 dpi. (F) SVV DNA levels in bronchial alveolar lavage (BAL) samples in all four monkeys as assessed by quantitative real-time PCR. SVV was first detected in BAL as early as 3 dpi, peaked 3–7 dpi and was not detected 17 dpi.

Since VZV is transmitted through aerosolized droplets, we inoculated four SVV-seronegative rhesus macaques (RM) once intrabronchially with 4×10^5^ pfu SVV. All four animals developed varicella either at 7- (24953 and 25043) or 10 (23942 and 23986) days post infection (dpi, Figure 1A). The rash progressed until 14 dpi after which lesions began to heal and resolved by 21 dpi. The severity of the rash varied between animals with 23986 displaying the greatest number of lesions followed by 24953, 23942 and 25043. Skin punch biopsies of the lesions confirmed the presence of both SVV DNA (Figure 1B) and SVV antigen (Figure 1C and D). There was considerable variation in viral loads in the lesions between animals and within the same animal (Figure 1B). This variation could be due to differences in the stage of the lesion. Skin biopsies obtained before infection were negative for both SVV DNA (Figure 1B) as well as SVV antigen (data not shown).

SVV DNA loads in peripheral blood mononuclear cells (PBMCs) (Figure 1E) and bronchial alveolar lavage (BAL) samples (Figure 1F) were determined by quantitative real time PCR (qPCR) using primers and probes specific for SVV ORF 21 (Table 1). SVV DNA was detected in PBMCs at 7 dpi, with a peak at 10 dpi and undetectable by 21 dpi, whereas BAL samples revealed SVV DNA first at 3 dpi, with a peak at 14 dpi and returning to undetectable levels at 17–21 dpi. Viral DNA loads were significantly higher (10^2^–10^5^ copies/100 ng) in BAL than in PBMCs (2–8 copies/100 ng), likely reflecting the route of infection. Monkeys were euthanized at 73 dpi (24953 and 25043) or 105 dpi (23942 and 23986) and all lymphoid tissues, lung, liver, gastrointestinal tract, pancreas, salivary glands, kidney, thyroid, dorsal root and trigeminal ganglia, brain and spinal cord were examined for the presence of SVV DNA by qPCR. SVV DNA was detected only in ganglia, but not in any other organ, including lung or liver (Table 2 and data not shown), indicating latent infection. Interestingly, the SVV viral loads in the ganglia correlated with the rash severity during acute infection with 23986 displaying the most extensive rash and 25043 the least.

**Table 1:** Primers and probes specific for the SVV ORFs used in real-time PCR.

ORF^a^	Direction	Sequence (5′–3′)	5′-location^b^
21	Forward	GACACATCAGCGGTTTGCA	32406
	Reverse	TGCACGCTGTGTTAGAATTCG	32541
	Probe	TCCATCCTGAACGATAGGCATGTCATAAAGA	32517
29	Forward	CGTCTTATAGGGCTGGCAAGC	54451
	Reverse	CTAACGCGCCATGAAGCAT	54520
	Probe	CGTATCAACGGCAAAATAAACGCGTGG	54473
40	Forward	GGATGCGTTATTGACGCTTCA	74906
	Reverse	TCCAGCATCCGGAGCTATTG	74993
	Probe	CGGATATGGCAGAACGTACCACTCCAA	74938
61	Forward	ACACAGCGCTAATGAGAAGCC	104077
	Reverse	GAAAGACGCTGCTGTTGTCG	104012
	Probe	CAACCCCGCGTGTTGGCCC	104051
62	Forward	CGATGAGCAGGCGGTATGA	107326
	Reverse	GGTAGGCCATGGTGGCATAA	107246
	Probe	CGCCCAAAAACATTTCATACTGCAAAGTTTAA	107306
63	Forward	CGTACGCTCCGCTGACAAA	111144
	Reverse	TGCTGTCCAATGCGTTTCTG	111206
	Probe	CGTCCCCGCACAATTACAGCGC	111164
66	Forward	TTGCTACTAACGCACCGGAA	113637
	Reverse	CCTGCGCTCCAAATATCGAC	113706
	Probe	CAAGGGATCCATACGGCCCGG	113664

a Numbers designate the SVV ORFs.

b Numbers designate the location of the 5′-end of the primers on the SVV genome (Genbank accession number AF275348).

**Table 2:** SVV DNA content in ganglionic and non-ganglionic tissues from rhesus macaques inoculated intrabronchially with SVV assessed by qPCR.

Monkey number	dpi^a^	Tissue	SVV DNA copies/ug of DNA	GAPdH
23942	105	Trigeminal	12	pos
		Cervical	0	pos
		Thoracic	5	pos
		Lum/sac^b^	24	pos
		Lung	0	pos
		Liver	0	pos
23986	105	Trigeminal	102	pos
		Cervical	163	pos
		Thoracic	182	pos
		Lum/sac^b^	40	pos
		Lung	nd^c^	nd^c^
		Liver	0	pos
24953	73	Trigeminal	16	pos
		Cervical	14	pos
		Thoracic	18	pos
		Lum/sac^b^	6	pos
		Lung	0	pos
		Liver	0	pos
25043	73	Trigeminal	0	pos
		Cervical	0	pos
		Thoracic	6	pos
		Lum/sac^b^	0	pos
		Lung	0	pos
		Liver	0	pos

a Days post-infection.

b Pooled ganglia from the lumbar and the sacral regions.

c nd  =  not done.

In latently infected human ganglia, transcripts specific for VZV ORFs 21, 29, 62, 63 and 66 are present [27]. To determine if SVV latency has a similar transcriptional pattern, SVV DNA-positive ganglia were further analyzed for transcripts corresponding to SVV ORFs 21, 29, 61 62, 63 and 66, as well as ORF 40, a major capsid protein that is expected to be absent during latency (primer and probes listed in Table 1). Transcripts specific for SVV ORFs 61, 21, 62, 63 and 66 were detected (Table 3). As expected SVV ORF 40 specific transcripts were not detected SVV ORF 29-specific transcripts were also not found (Table 3). VZV ORF 29 (a single-stranded DNA-binding protein) -specific transcripts are sometimes present and sometimes absent in latently infected human ganglia. SVV ORF 61 specific transcripts were the most abundant (Table 3). Both sense and antisense transcripts of SVV ORF 61 were detected, and antisense transcripts were 5–9 times more abundant than sense transcripts (Table 4), consistent with findings of Ou et al. [28]. No transcripts were detected in control PCR reactions in the absence of reverse transcriptase. We routinely detect as little as one copy of the SVV ORFs listed above in positive controls. Thus, as previously described for VZV, SVV latency is associated with limited viral transcriptional activity.

**Table 3:** Abundance of SVV-specific transcripts in SVV DNA-positive ganglia (per 1–3 µg RNA) assessed by qRT-PCR.

Monkey number	Tissue	SVV ORFs						
		21	29	40	61	62	63	66
23942	Trigeminal	0	0	0	217	0	0	0
	Thoracic	0	0	0	52	0	2	0
	Lum/sac	0	0	0	389	trace	0	0
23986	Trigeminal	3	0	0	807	0	7	0
	Cervical	4	0	0	2500	0	9	0
	Thoracic	0	0	0	2700	5	6	0
	Lum/sac	0	0	0	5300	3	18	0
24953	Trigeminal	0	0	0	51	0	0	0
	Cervical	0	0	0	55	0	0	0
	Thoracic	0	0	0	600	6	6	3
	Lum/sac	0	0	0	8	0	0	0
25043	Trigeminal	0	0	0	10	0	0	0
	Cervical	0	0	0	6	0	0	0
	Thoracic	0	0	0	21	0	0	0
	Lum/sac	0	0	0	0	0	0	0

**Table 4:** Abundance of SVV ORF 61 sense and antisense transcripts (per 1–3 µg RNA) in SVV DNA-positive ganglia assessed by qRT-PCR.

Monkey number	Tissue	SVV ORF 61 PRIMER used in RT	
		61R (antisense)^a^	61F (sense)^b^
23942	Trigeminal	25	153
	Thoracic	14	62
	Lum/Sac	80	584
23986	Trigeminal	527	7300
	Cervical	1277	8609
	Thoracic	1135	9835
24953	Cervical	8	53
	Thoracic	148	4100
	Lum/Sac	0	0
SVV-infected Vero		1×10^7^	5×10^5^

a Detects sense transcripts.

b Detects antisense transcripts.

**Figure 2:**
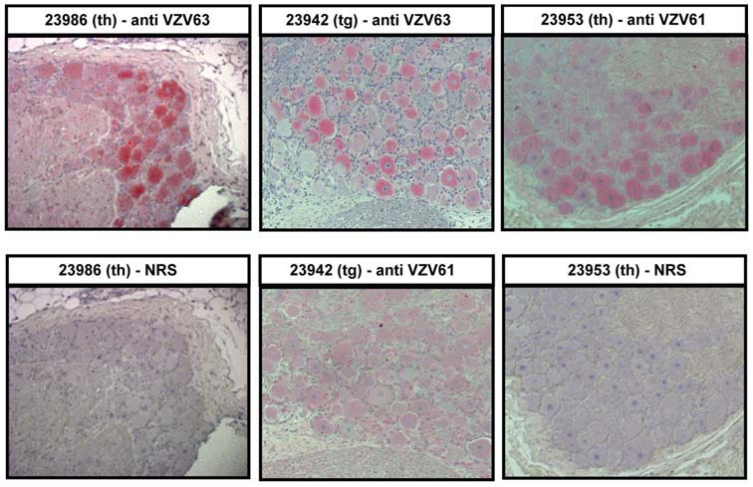
Detection of SVV ORF 63 protein in the cytoplasm of neurons in monkey ganglia latently infected with SVV. Paraformaldehyde-fixed, paraffin-embedded sections (5 µ) of thoracic (th) ganglia from monkey 23986, trigeminal (tg) ganglia from monkey 23942 and thoracic ganglia from monkey 24953 were analyzed by immunohistochemistry using a 1∶800 dilution of rabbit anti-VZV ORF 63 (anti-VZV63), a 1∶1000 dilution of rabbit anti-VZV ORF 61 (anti VZV61) antiserum or normal rabbit serum (NRS). SVV ORF 63 protein was detected in thoracic and trigeminal ganglia of monkeys 23986 and 23942, respectively, and SVV ORF 61 protein was detected in thoracic ganglia of monkey 24953. No SVV antigen was seen using normal rabbit serum. SVV ORF63 protein, but not SVV ORF61 protein was detected in trigeminal ganglia of monkey 23942.

VZV ORF 63 protein is present in the cytoplasm of neurons in latently infected human ganglia [29],[30]. To determine whether SVV ORF 63 protein is comparably localized, sections of formalin-fixed ganglia from the side of the neuraxis opposite to the one used for nucleic acid analysis were studied using immunohistochemistry for SVV ORF 63 protein as well as for SVV ORF 61 protein in light of the considerable abundance of SVV ORF 61 transcripts. VZV ORF 63- or 61-specific rabbit polyclonal antisera, which reacted with SVV-infected cells in culture (Figure S1A, C) but not with uninfected Vero cells (Figure S1B, D), were used in these studies. We detected SVV ORF 63 protein exclusively in the cytoplasm of ganglionic neurons in all animals (Figure 2A and B). SVV ORF 61 protein was detected in one ganglion from two monkeys (Table 5) in both the nucleus and cytoplasm (Figure 2). Analysis of adjacent sections of trigeminal ganglia from monkey 23942 revealed the presence of SVV ORF 63 but not 61 (Figure 2). Table 5 summarizes results from ganglia of all 4 monkeys and shows that like VZV ORF63, SVV ORF63 is consistently detected in the cytoplasm of neurons in latently infected ganglia. Overall, these data suggest that SVV infection in rhesus macaques recapitulates key features of VZV latency.

**Table 5:** Detection of SVV antigens in latently infected rhesus macaque ganglia.

Monkey number	Tissue	SVV antigens (ORFs)	
		61	63
23942	Trigeminal	−	+
	Lum/sac	−	+
23986	Trigeminal	−	+
	Thoracic	−	+
24953	Trigeminal	−	nd^a^
	Thoracic	+	+
	Cervical	−	+
	Lumbar	−	+
25043	Trigeminal	−	+
	Thoracic	−	+
	Cervical	+	+
	Lumbar	−	+

a nd  =  not done.

### Rhesus macaques inoculated with SVV generate a B cell response

Resolution of VZV infection in humans correlates with the development of humoral and cellular immune responses to virus [31]. A hallmark of the antiviral immune response is expansion of antigen-specific T and B cells, which can be measured by flow cytometry [32] based on expression of Ki67, a nuclear protein involved in DNA replication. To determine whether SVV infection in rhesus monkeys elicits similar immune responses as VZV, we measured the magnitude and kinetics of the B and T cell responses after the appearance of rash.

**Figure 3:**
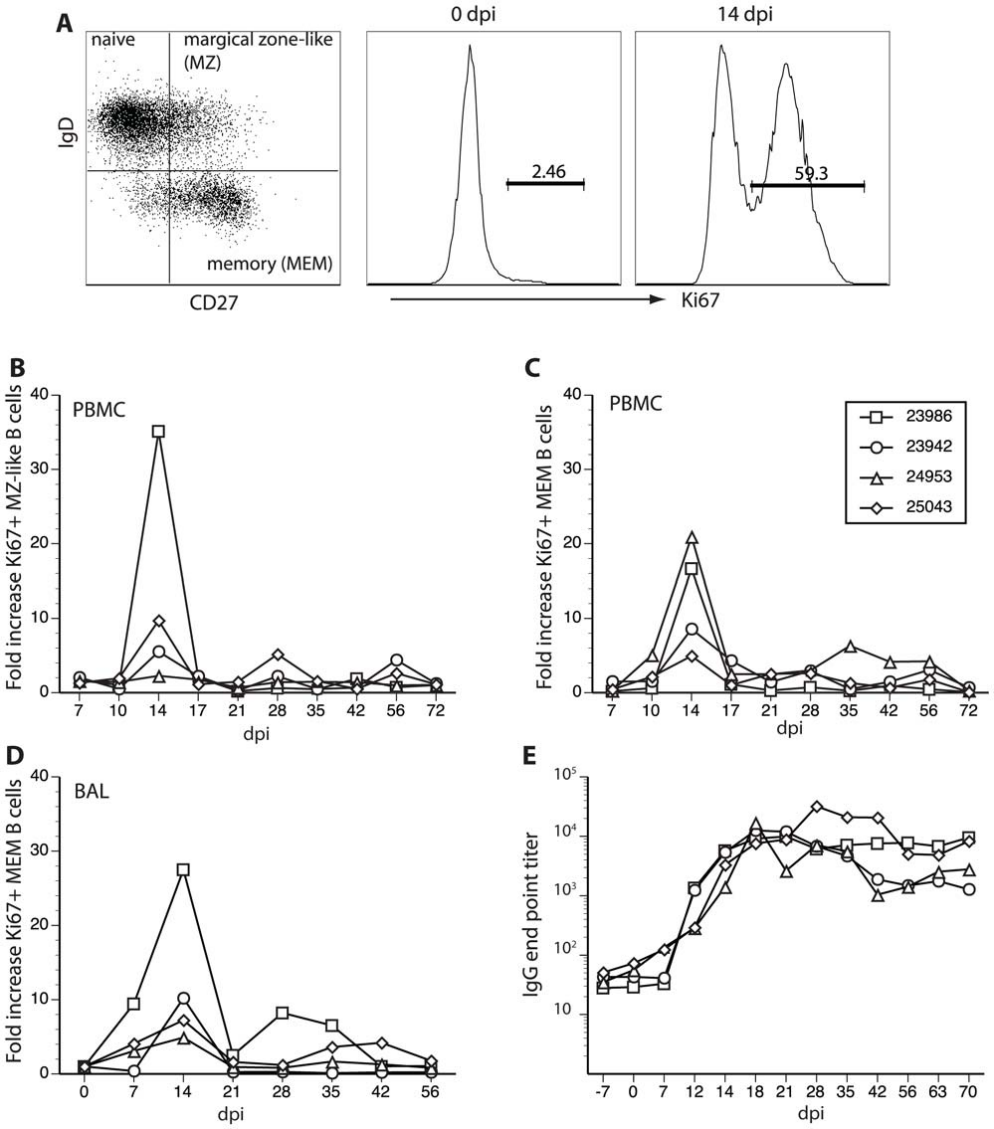
SVV infection of rhesus macaques elicits a B cell response after the appearance of varicella rash. B cells were separated into three subsets based on expression of IgD and CD27: CD27−IgD+; CD27+IgD+; and isotype-switched CD27+IgD− cells. A representative profile from PBMCs obtained from monkey 24953 on day 0 (A, left) is shown. B cell proliferation within CD27+IgD+ and CD27+IgD− subsets was measured using flow cytometry based on expression of the nuclear protein Ki67, which is up-regulated upon entry into the cell cycle. B cell proliferation was minimal before SVV infection (0 dpi, A, middle), but increased dramatically 14 dpi (A, right). Throughout the course of SVV infection in all 4 monkeys, the number of Ki67+ CD27+IgD+ (panel B) and CD27+IgD− B cells (C) increased dramatically 14 dpi compared to 0 dpi in PBMCs. In BAL of all 4 monkeys, increased numbers of Ki67+ IgD−CD27+ B cells were seen throughout the course of infection compared to 0 dpi (panel D), although no significant proliferation of IgD+CD27+ B cells was detected (data not shown). In all 4 monkeys, SVV-specific IgG antibodies appeared 7 dpi, peaked 18–21 dpi (as detected by ELISA) and remained stable up to 70 dpi (E).

To assess the B cell response, we measured the fold increase in the number of Ki67+ isotype-switched B cells. PBMCs and BAL cells were stained with antibodies directed against CD20, IgD and CD27 to distinguish between three subsets: CD27-IgD+ (most likely naïve), CD27+IgD+, and CD27+IgD− (isotype-switched B cells) [33] (Figure 3A, left). Cells were then fixed and the nuclear membrane was permeabilized before staining with anti-Ki67 antibody (Figure 3A, middle and right). SVV infection induced a robust proliferation of CD27+IgD+ as well as isotype-switched CD27+IgD− B cells in PBMCs as indicated by an increase in the frequency of Ki67+ cells on dpi 14 compared to dpi 0 (Figure 3A middle and right panels; Figure 3B and C, respectively). Analysis of BAL samples revealed an increased frequency of proliferating CD27+IgD− B cells, that were detected earlier (7 dpi) than in PBMCs (14 dpi) (Figure 3D). No significant proliferation of CD27+IgD+ B cells was found (data not shown). The SVV-specific IgG titer revealed an IgG response by 12 dpi that peaked18–21 dpi (Figure 3E).

### Rhesus macaques inoculated with SVV generate a T cell response

**Figure 4:**
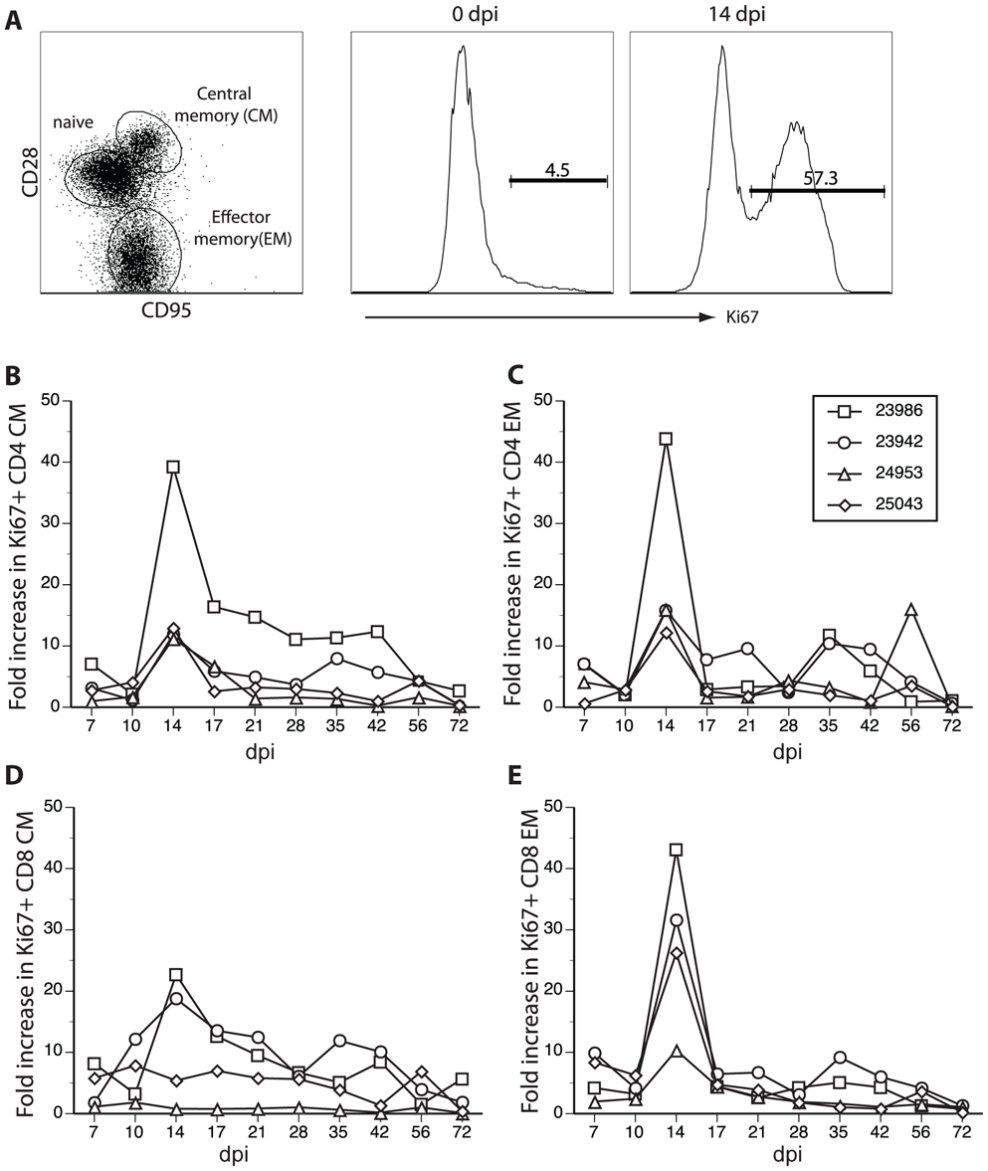
SVV infection of rhesus macaques elicits a robust peripheral T cell response. CD4 and CD8 T cells were separated into three subsets based on expression of CD28 and CD95: naïve (CD28+CD95−); central memory (CM, CD28+CD95+); and effector memory (EM, CD28−CD95+) cells. A representative profile of CD8 T cell subsets of monkey 24953 on 0 dpi is shown (A, left). T cell proliferation was measured as described in Figure 3 based on expression of the nuclear protein Ki67 using flow cytometry. T cell proliferation dramatically increased 14 dpi (A, right) compared to 0 dpi (A, middle). Fold increases (relative to 0 dpi) in the numbers of Ki67+ CD4 CM and EM (B and C) and CD8 CM and EM T cells (D and E) in PBMCs indicate a peak proliferative response of all T cell subsets 14 dpi in all monkeys (B–E).

**Figure 5:**
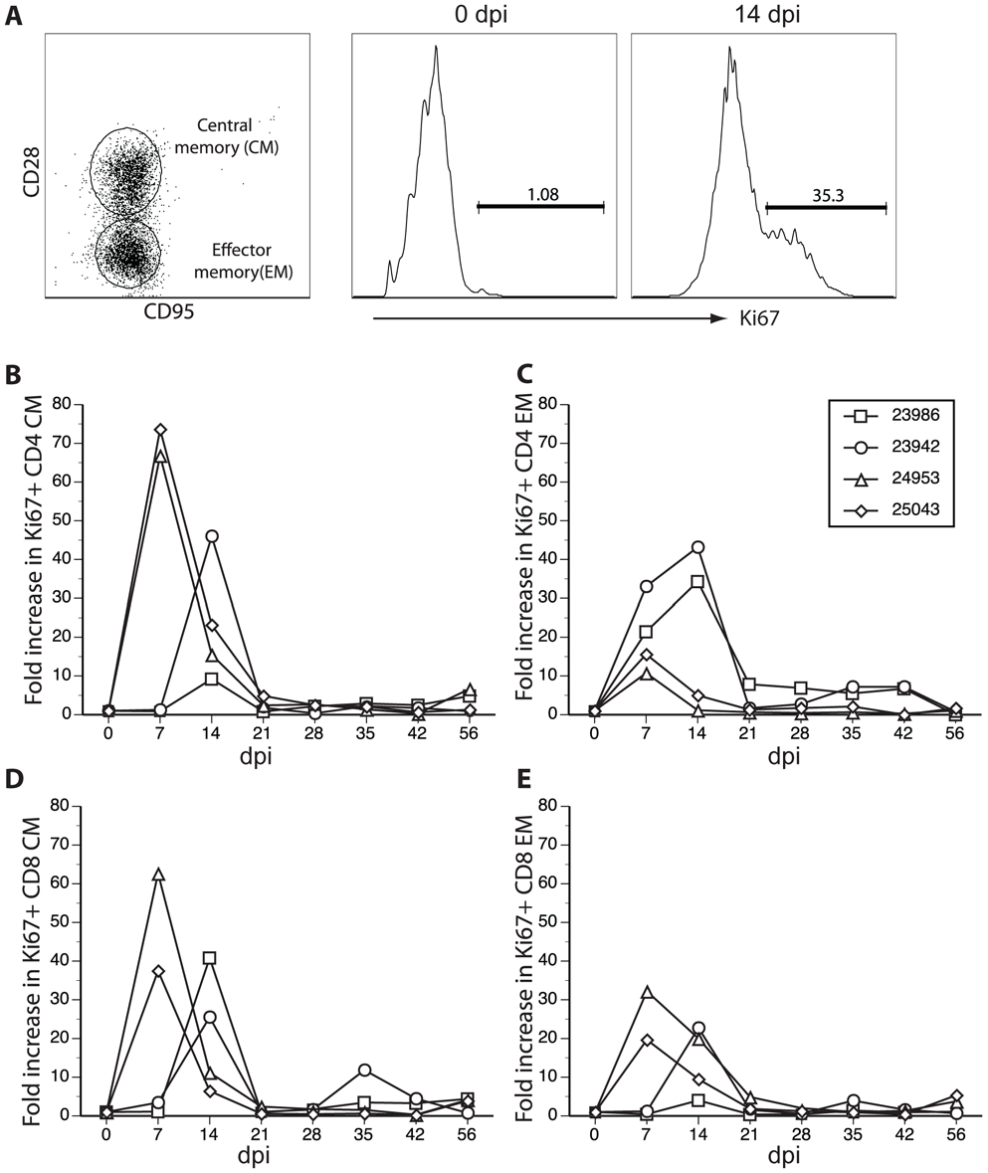
SVV-induced T cell proliferation occurs earlier in BAL than in peripheral blood. In contrast to PBMCs, BAL revealed only CM and EM T cells subsets as illustrated by a profile of CD8 T cells from 24953 day 0 (A, left). T cell proliferation was measured as described in Figure 3. A representative example of Ki67 staining within CD8 EM T cells from monkey 24953 on 0 dpi (A, middle) and 14 dpi (A, right) is shown. Fold increases (relative to 0 dpi) in the percentages of Ki67+ CD4/CM, CD4/EM, CD8/CM and CD8/EM T cells subsets in BAL from all 4 monkeys indicate a robust proliferative response of all T cell subsets 7–14 dpi in all monkeys (B–E).

T cell proliferation in PBMCs (Figure 4) and BAL (Figure 5) was also determined by measuring the relative (fold) increase in the number (PBMC) or percentage (BAL) of Ki67^+^. At each time point, PBMCs and BAL cells were stained with antibodies directed against CD4, CD8, CD28 and CD95 (Figure 4A and 5A, left) to delineate naïve (CD28+CD95−), central memory (CD28+CD95+, CM) and effector memory (CD28−CD95+, EM) T cell subsets [34]. Cells were fixed, permeabilized and incubated with anti-Ki67 antibodies (Figures 4A and 5A, middle and right). Previous studies showed that naïve T cells identified using these markers were CCR7+. EM T cell were CCR7-, whereas CM T cells contained a transitional population that lacked CCR7 expression [32],[35]. Both naïve and memory T cells were detected in PBMCs (Figure 4A, left), whereas, as expected, only memory T cells were present in BAL cells (Figure 5A, left). SVV infection induced robust T cell proliferation as shown by an increase in Ki67+ CD8 EM T cells (Figures 4A and 5A, middle and right).

SVV infection resulted in proliferation of both CM and EM T cell subsets in PBMCs that peaked at 14 dpi in all 4 monkeys (Figure 4A, right; Figure 4B–E). In BAL cells, CD4 EM T cell proliferation was detected at 7 dpi in all animals (Figure 5C). However, proliferation of other T cell subsets in BAL was detected at 7 dpi in monkeys 24953 and 25043 and 14 dpi in monkeys 23986 and 23942 (Figure 5B, D and E). Interestingly, varicella rash was detected slightly earlier in monkeys 24953 and 25043 (day 7) compared to monkeys 23986 and 23942 (day 10), which could explain the earlier proliferative response observed in monkeys 24953 and 25043. Furthermore, 25043 had the lowest viral load, least amount of rash and lowest T cell proliferation, which suggests that T cell proliferation correlates with viral load. Overall, as described for B cells, T cell proliferation occurred earlier in BAL compared to peripheral blood.

**Figure 6:**
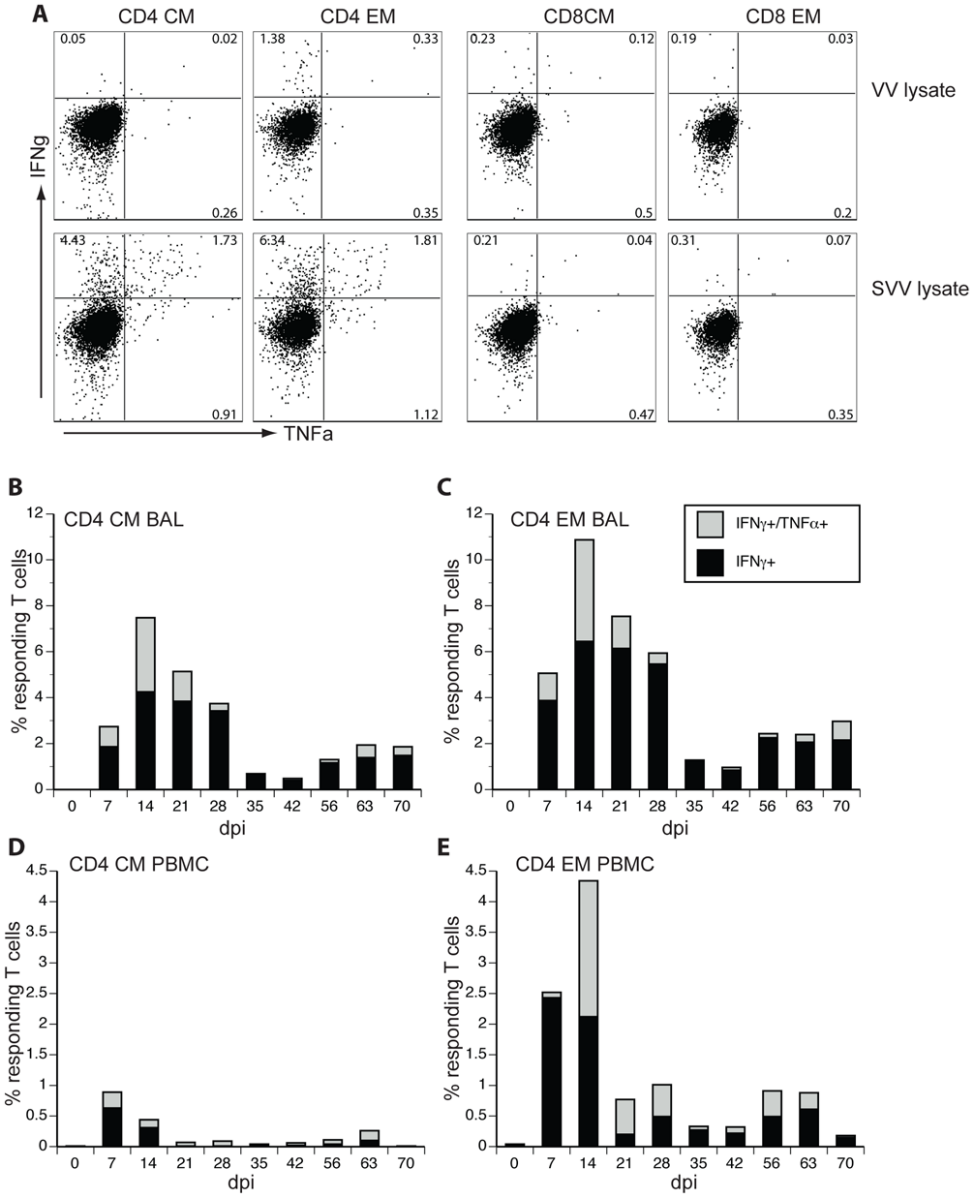
Appearance of SVV-specific CD4 T cells correlates with Ki67 expression. The frequency of SVV-specific T cells in PBMCs and BAL was measured by intracellular cytokine staining. (A) Representative examples of IFNγ and TNFα production by CD4 CM, CD4 EM, CD8 CM and CD8 EM T cells from BAL of monkey 24953 7 dpi after stimulation with vaccinia virus (VV) lysate (negative control) and SVV lysate. A robust response by CD4 CM and CD4 EM cells was detected after exposure to SVV lysates, but not VV lysates (A, left), whereas no response by CD8 CM and CD8 EM cells was seen after exposure to either SVV or VV lysate (A, right). The percentage of responding (IFNγ+ alone or both IFNγ+ and TNFα+) CD4 CM and CD4 EM T cells in BAL (B and C) and PBMCs (D and E) in all 4 monkeys was determined and averaged. At all time points, more SVV-responsive CD4/CM and CD4/EM cells were found in BAL (B and C) than in PBMCs (D and E).

To determine the frequency of SVV-specific T cells, we used intracellular cytokine staining analysis to detect IFNγ/TNFα-producing T cells after stimulation with SVV antigen. Since SVV is highly cell-associated and cell-free SVV is difficult to obtain, we used an SVV lysate (1 μg/well) to stimulate PBMCs and BAL cells isolated from infected monkeys at different dpi in the presence of Brefeldin A, which blocks protein export. This allowed efficient detection of cytokine-producing CD4 cells, which responded to SVV but not to the vaccinia (VV) lysate (Figure 6A); CD8 T cells showed no detectable response to either lysate (Figure 6A, right), likely due to the absence of free virus in the SVV lysate; another possibility is a low efficacy of cross-presentation of MHC class I-associated viral antigens due to the low abundance of viral proteins compared to cellular proteins in the lysate. Previous studies with VZV viral lysates did not detect CD8 T cell responses ex vivo without prior stimulation [36]. SVV-specific CD4 T cells were detected at 7 dpi, with a peak at 14 dpi in both BAL (Figure 6B and C) and PBMCs (Figure 6D and E), but the frequency of responding CD4 CM and EM T cells was higher in BAL compared to PBMCs. After 14 dpi, the frequency of responding CD4 T cells decreased and a stable memory population was established.

**Figure 7:**
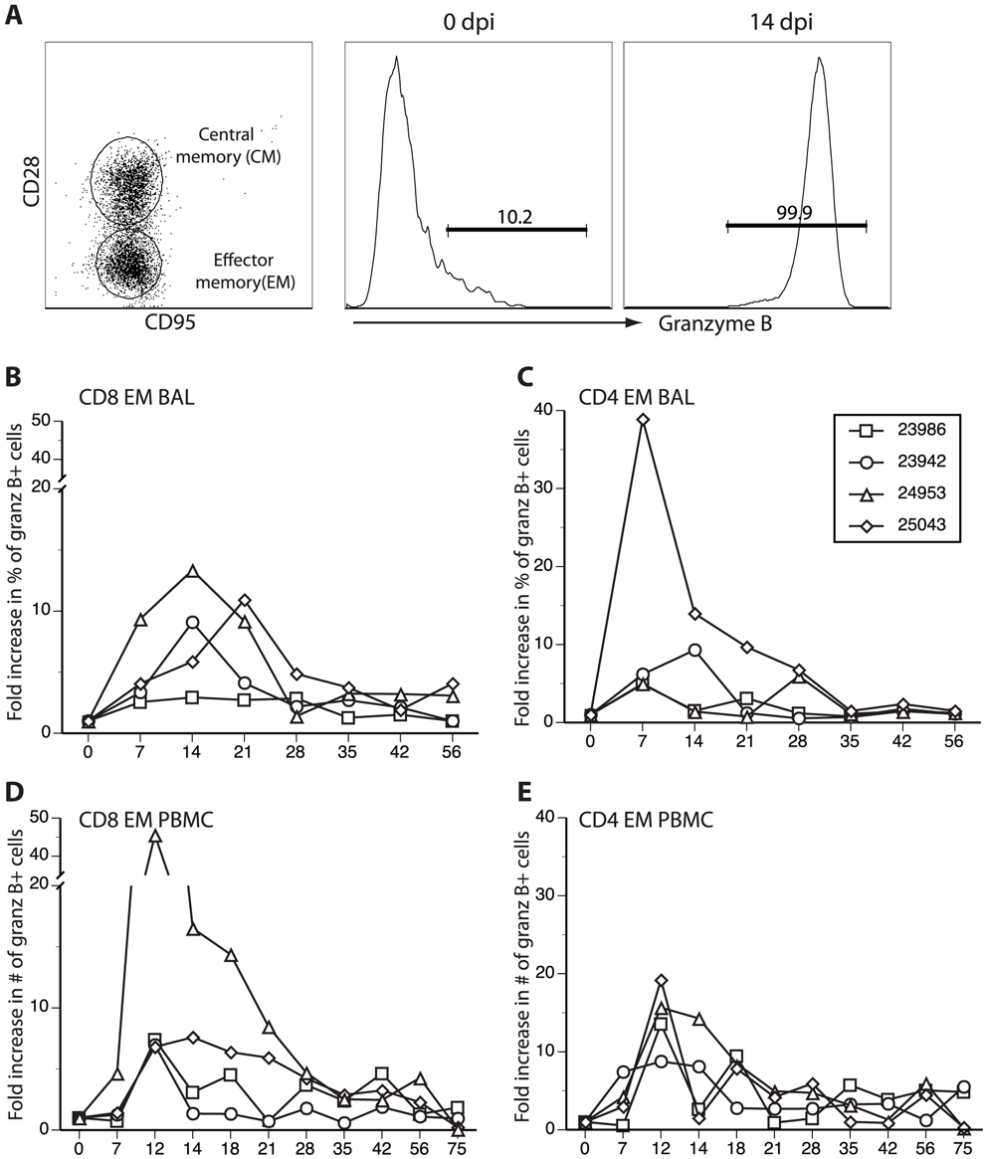
Increased granzyme B expression in EM T cells after SVV infection. (A) Intracellular granzyme B expression in CD4 and CD8 T cell subsets as assessed by flow cytometry. An example of CD8 T cell subsets in BAL on day 0 from animal 24953 is shown in left panel. Frequency of granzyme B+ EM T cells increased dramatically after SVV infection as shown in BAL-resident CD8 EM T cells from monkey 24953 (A, middle and right), while the increase in granzyme B+ CM T cells was comparatively modest (data not shown). Measurement of fold increases (compared to 0 dpi) in the percentages of granzyme B-expressing CD8/EM and CD4/EM from BAL and PBMCs during the course of infection indicated a significant increase in these percentages in both BAL and PBMCs 12–14 dpi in all samples (B–E).

Since VZV infection is associated with the development of cytolytic CD4 and CD8 T cells [37],[38],[39], we measured an increase in granzyme B, a major component of cytolytic granules and an indicator of cytolytic activity. A considerable increase in granzyme B+ EM CD8 and CD4 T cells in both BAL (Figure 7B and C) and PBMCs (Figure 7D and E) was found in all samples as compared to levels at 0 dpi (Figure 7A, middle). These data indicate that SVV infection elicits a T cell response with cytotoxic potential.

## Discussion

In this study, we show for the first time that intrabronchial infection of rhesus macaques with SVV results in viremia and varicella rash followed by resolution of viremia and establishment of SVV latency in ganglionic neurons. The incubation period for SVV (7–10 days) seems to be slightly shorter than that of VZV, which has been estimated at 14 days. As early as 2 months after varicella, SVV DNA was found exclusively in ganglia and not in any of multiple visceral tissues examined. Transcripts corresponding to SVV ORFs 21, 61, 62, 63 and 66, but not 40 or 29, were also found in latently infected ganglia (Table 3). Except for ORF 61, the SVV transcripts found in ganglia are the same as those detected in latently infected human ganglia [27]. The detection of SVV ORF 61 RNA in latently infected monkey ganglia suggests that it should be searched for in human ganglia latently infected with VZV. Finally, like VZV, SVV ORF 63 protein was seen exclusively in the cytoplasm of neurons in latently infected monkey ganglia.

Two different NHP models of SVV infection have been developed. African green or Cynomolgus monkeys inoculated intratracheally with SVV develop viremia followed by varicella and an antibody response [23],[40],[41]. Unfortunately, this model does not results in latency but rather the persistence of SVV DNA in blood, lung and liver for months to years after experimental infection [24],[25]. More recently, Mahalingam et al., developed a natural infection model whereby naïve African green or Cynomolgus monkeys were exposed to experimentally inoculated cage mates [21],[26]. Although naturally infected animals developed varicella followed by the establishment of latency, viremia and seroconversion were not consistently observed [21],[26]. Thus, in our initial experiments we inoculated two rhesus macaques intravenously. The first monkey developed persistent viremia. The second monkey was re-challenged with SVV 4 months later, at which point it did not show any signs of disease and SVV DNA was detected only on 4 dpi, indicative of subclinical infection. These data suggested that the immune response generated after primary infection protects against subsequent exposures. Encouraged by the results in the latter monkey and the fact that varicella virus is naturally transmitted through inhaled aerosolized viral droplets, we redesigned our strategies to deliver virus by the intrabronchial route. This strategy led to viremia, varicella, the establishment of latency in sensory ganglia, and the development of SVV-specific cellular and humoral immune responses. Although the difference in the route of infection (intratracheal versus intrabronchial) cannot be discounted, it is very likely that the change in nonhuman primate species is the main reason for resolution of viremia. For example, African green monkeys infected with simian immunodeficiency virus (SIV) show high levels of viremia without signs of simian AIDS. In contrast, rhesus macaques infected with SIV develop a profound loss of CD4 T cells and simian AIDS [42],[43].

As described for VZV [44],[45],[46], SVV infection resulted in viremia and in the detection of SVV DNA in T cells (CD4, CD8 and γδ), B cells, macrophages and dendritic cells (data not shown). The increased levels of SVV DNA in BAL cells compared to PBMCs are consistent with the severity of VZV infection seen in lungs of humans with varicella [47],[48],[49],[50]. The presence of SVV ORF61 transcripts was the only difference between VZV and SVV latency. SVV ORF 61 protein shares 37% amino acid identity with that of VZV ORF 61 protein with highly conserved domains [51]. Transcripts associated with VZV ORF61 have not been reported in latently infected human ganglia. Nevertheless, given parallels between varicella latency in humans and monkeys, a more aggressive search for VZV ORF 61 transcripts in humans is indicated. Interestingly SVV ORF 61 protein was detected only sparingly despite the presence of a high level of transcripts. It is unlikely that the low level detection of SVV ORF61 is due to poor sensitivity of the antibody used. Rather, the high number of antisense transcripts detected in same ganglia could be inhibiting protein translation.

The kinetics of the immune response to SVV in rhesus macaques also parallel findings in humans infected with VZV. In humans, antibodies are detected at about 6 days after the onset of varicella rash, and we found peak SVV-specific IgG titers about 8 days after rash (18 dpi). VZV-specific T cells are present at 3 days after the onset of varicella in humans [52]. We detected T cell proliferation after SVV infection at 7 dpi, with a peak at 14 dpi, i.e., at about 4 days post-rash, and SVV-specific IFNγ-secreting T cells were also first detected at 7 dpi and peaked at 14 dpi. We also detected an increase in granzyme B+ T cells indicative of a cytotoxic T cell response as described for VZV [53]. In peripheral blood, the peak of the proliferative burst occurred 14 dpi with 23986 displaying the highest level of proliferation in all T cell subsets analyzed. Interestingly, 23986 displayed the most extensive rash and the highest viral load in ganglia. On the other hand, monkey 25043, which had the least amount of rash, viral DNA and minimal virus transcription in latently infected ganglia, also had a low proliferative T cell response. The kinetics of T cell proliferation in BAL correlated with the onset of the rash, with T cells form 24953/25043, which showed signs of varicella on day 7, proliferating earlier (7 dpi) that T cells from 23942/23986, which showed sings of varicella on day 10. These data suggest a correlation between the extent of rash, the development of the T cell response and the extent of viral load and expression in latently infected ganglia. Overall, SVV-infected rhesus macaques develop an adaptive immune response to virus with kinetics like those seen in humans after primary infection with VZV.

Together, our data indicate that SVV infection of rhesus macaques provides a robust animal model that recapitulates clinical, virological and immunological hallmarks of VZV infection in humans. This model will provide unique opportunities to define host and viral factors needed to maintain latency and to characterize immune correlates needed for protection against VZV infection. Future studies will dissect the antiviral functions of the antibody response (neutralization, complement fixation, ADCC). It will also be important to characterize the specificity and function of SVV-specific CD4 and CD8 T cells, and to determine if the SVV homologues of VZV ORFs 4, 10, 29, 62, 63, gI and gE are also immunogenic in rhesus macaques [39],[54],[55],[56],[57].

## Materials and Methods

### Ethics statement

All animals were handled in strict accordance with good animal practice as defined by the relevant national and/or local animal welfare bodies, and all animal work was approved by the Oregon National Primate Research Center Institutional Animal Care and Use Committee (IACUC#0779).

### Cells and virus

The Delta strain of SVV was propagated in Vero cells as described [17]. SVV cell lysate were obtained by scraping SVV-infected Vero cells at the height of a cytopathic effect (CPE). Infected cells were pelleted by centrifugation at 500G for 5 min and resuspended in RPMI medium and sonicated using 7 pulses of 70–80 W (Sonicator 3000, Misonix) followed by centrifugation at 100G for 5 min. Vaccinia virus (VV) WR strain lysates were obtained from Vero cells infected at a multiplicity of infection of 0.1, harvested by scraping at the height of CPE, and treated as described for SVV-infected cells. Total protein concentration was determined by standard Bradford assay (Bio-Rad, Hercules, CA). 1 ug/well was used in T cell stimulation assays.

### Monkeys

Four 3-to-4-year-old male rhesus macaques (*Macaca mulatta*), seronegative for SVV and without any history of chronic illness, were inoculated intrabronchially with 4×10^5^ PFU SVV. Rhesus macaques were sedated by ketamine (10 mg/Kg) and positioned in dorsal recumbency and monitored continuously with a pulse oximeter. A pediatric fiber optic endoscope was introduced into the tracheal lumen with the aid of a laryngeal scope followed by instillation of 1 ml of 1% lidocaine to control bronchospasm. The tip of the bronchoscope was gently wedged into a right subsidiary bronchus, and the virus inoculum was infused in a volume of 2 ml followed by additional infusion of a 5 ml aliquot of sterile, pyrogen-free saline into the bronchus. Heparinized blood and bronchial alveolar lavages (BAL) were obtained after ketamine (10 mg/kg body weight) sedation. PBMCs were isolated using a histopaque gradient (Sigma) as per the manufacturer's recommendation. The absolute number of lymphocytes/μl blood was obtained using a complete blood count machine (Hemavet, Drew Scientific). BAL samples were pelleted and resuspended in RPMI medium supplemented with 10% FBS, penicillin/streptomycin and L-glutamine. During varicella, monkeys were anesthetized and a punch biopsy of the area of rash was obtained and divided into two portions; one portion was used to extract total DNA, and the second portion was fixed in 4% paraformaldehyde and paraffin-embedded. Monkeys were euthanized at 73 or 105 dpi and ganglionic and non-ganglionic tissues were harvested at necropsy. Tissues were divided into two portions; one portion was snap-frozen for DNA extraction and the other portion was fixed in 4% paraformaldehyde and paraffin-embedded. Ganglia from the two sides of the neuraxis were kept separate. Pooled ganglia from specific dermatomes and from one side of the neuraxis were snap-frozen in liquid nitrogen, and pooled ganglia from the same dermatomes of the other side were fixed in 4% paraformaldehyde and paraffin-embedded.

### DNA/RNA extraction and real-time PCR

DNA was extracted from heparinized whole blood, from skin biopsies and from BAL as well as from portions of frozen lung and ganglia using the Qiagen genomic DNA extraction kit (Qiagen) as described [58]. RNA was extracted from SVV DNA-positive ganglia as previously described [59]. SVV DNA loads were determined by real-time PCR using primers and probes specific for ORF 21 using the ABI 7700 and ABI StepOne instruments (Applied Biosystems).

Oligonucleotide primers (Integrated DNA Technologies) and probes specific for SVV ORFs 21, 29, 40, 61, 62, 63 and 66 were used to amplify and detect SVV DNA and RNA in monkey tissues. Table 1 lists the primer sequences and probes used. Total RNA (1 µg) was treated with 1 U of DNase (Invitrogen) for 15 min at room temperature in a 10-µl volume The reaction was terminated by the addition of 1 µl 25 mM EDTA and heating at 65°C for 10 min and transferred to ice. Reverse transcription was performed using the Transcriptor First Strand cDNA Synthesis Kit (Roche) with anchored-oligo(dT)_18_ primers and random hexamer primers, as per manufacturer's instructions in a total volume of 20 µl. The reaction was then diluted to 50 µl with nuclease-free water.

Real-time PCR was performed in a 20-µl volume of 1X qPCR Mastermix (Abgene Products) containing 900 nM concentrations of each SVV ORF specific primer, 250 nM probe and 10% of each cDNA sample using the 7500-Fast real-time PCR system (Applied Biosystems). All samples were analyzed in triplicate. Amplification conditions included initial denaturation at 95°C for 15 min followed by 50 two-step cycles of 15 sec at 95°C and 1 min at 60°C. Sense and anti-sense transcripts specific for SVV ORF61 were analyzed using SVV ORF 61 reverse or forward primer (Table 1), respectively in the reverse transcription step.

### Measurement of T and B cell proliferation

PBMC and BAL cells were first stained with surface antibodies directed against CD8b (Beckman Coulter), CD4, CD28 and CD95 (Biolegend) to delineate naive, central and effector memory T cell subsets as described [32],[34],[60]. Cells were then fixed and permeabilized as per the manufacturer's recommendation using fixation buffer (Pharmingen). The nuclear membrane was subsequently permeabilized using fixation buffer supplemented with 10% DMSO, and cells were stained with an antibody directed against Ki67 as per the manufacturer's recommendation (Pharmingen). Samples were prepared using the LSRII instrument (Beckton Dickenson) and FlowJo software (TreeStar). The percentages of Ki67+ cells within T and B cell subsets were converted to absolute numbers using the lymphocyte numbers/μl whole blood obtained using a complete blood count machine (Hemavet). For instance, the number of Ki67^+^ CD8 EM T cells in PBMCs was calculated as: number of lymphocytes x percentage of CD8 T cells x percentage of EM CD8 T cells x percentage of Ki67^+^ cells within CD8 EM.

### Intracellular cytokine staining

PBMCs and BAL cells were stimulated with lysates from SVV (1 µg) or VV (negative control) or with CD3 (0.1 µg/sample, positive control) for 12 h followed by incubation with Brefeldin A (Sigma) for 6 h to block cytokine export. After stimulation, cells were stained with surface antibodies directed against CD4, CD8b, CD28 or CD95 as described for Ki67 staining above. Samples were then fixed and permeabilized using fixation buffer (Pharmingen) before addition of antibodies to detect IFNγ and TNFα (Biolegend). Samples were prepared on the LSRII and analyzed by FlowJo software as described above.

### Granzyme B staining

PBMCs and BAL were stained with surface antibodies directed against CD8b, CD4, CD28 and CD95 to delineate naive, central and effector memory T cell subsets as described above. Cells were then fixed and permeabilized using fixation buffer (Pharmingen) before the addition of anti-granzyme B antibody (Pharmingen). The number of granzyme B+ cells was calculated as: number of lymphocytes obtained using the complete blood count machine x percentage CD8 T cells x percentage EM cells x percentage granzyme B+ cells.

### SVV-specific enzyme-linked immunoabsorbent assay (ELISA)

ELISA plates were coated with SVV lysates (0.25 ug/ml) overnight at 4°C, washed 3 times with 0.05% Tween-PBS and incubated with 3-fold dilutions of plasma in triplicate for 1 h. After 3 additional washes with 0.05% Tween-PBS, plates were incubated with horseradish peroxidase (HRP)-conjugated anti-rhesus IgG (Nordic Immunology) for 1 h, followed by addition of chromogen OPD substrate to allow detection and quantitation of bound antibody molecules. End-point IgG titers were calculated using log-log transformation of the linear portion of the curve, and 0.1 OD units as cut-off. For each plate, a positive control sample (pooled plasma obtained from monkeys 23974 and 23986 at necropsy) was used to normalize ELISA titers among assays, and a negative control sample (pooled plasma from specific-pathogen-free rhesus macaques) was used to ensure specificity of the assay conditions.

### Immunohistochemistry

Sections (5 µm) of paraformaldehyde-fixed, paraffin-embedded skin, lung or ganglia were analyzed for SVV antigen expression by immunohistochemistry [21],[29] using: a 1∶800 dilution of normal rabbit serum; a 1∶2000 dilution of a mixture of polyclonal anti-peptide antibodies raised in rabbits against SVV gH and gL [61]; a 1∶1000 dilution of polyclonal anti-VZV ORF 61 protein [62]; or a 1∶800 dilution of anti-VZV ORF 63 protein [29]. All reagents were diluted in PBS containing 5% normal sheep serum and adsorbed using acetone-fixed uninfected Vero cells and acetone-fixed normal human liver powder. Sections from skin punch biopsies were stained using a 1∶500 dilution of normal rabbit serum or rabbit polyclonal antibodies against gH/gL diluted in PBS containing 5% normal sheep serum.

## Supporting Information

Figure S1 VZV ORF 63- and 61-specific antibodies cross-react with SVV ORF63 and 61 proteins in virus-infected cells. SVV-infected (A, C) and uninfected (B, D) Vero cells in culture were analyzed by immunohistochemistry using a 1∶800 dilution of rabbit anti-VZV ORF 63 (A, B) or a 1∶1000 dilution of rabbit anti-VZV ORF 61 antiserum (C, D). ORF 61 and 63 proteins were detected only in infected cells.
(3.49 MB TIF)
