# Supplementary figures and images for: Simian Varicella Virus Infection of Rhesus Macaques Recapitulates Essential Features of Varicella Zoster Virus Infection in Humans
Source: PLoS Pathog. 2009 Nov 13;5(11):e1000657. doi: 10.1371/journal.ppat.1000657 (PMC2770849; doi:10.1371/journal.ppat.1000657)

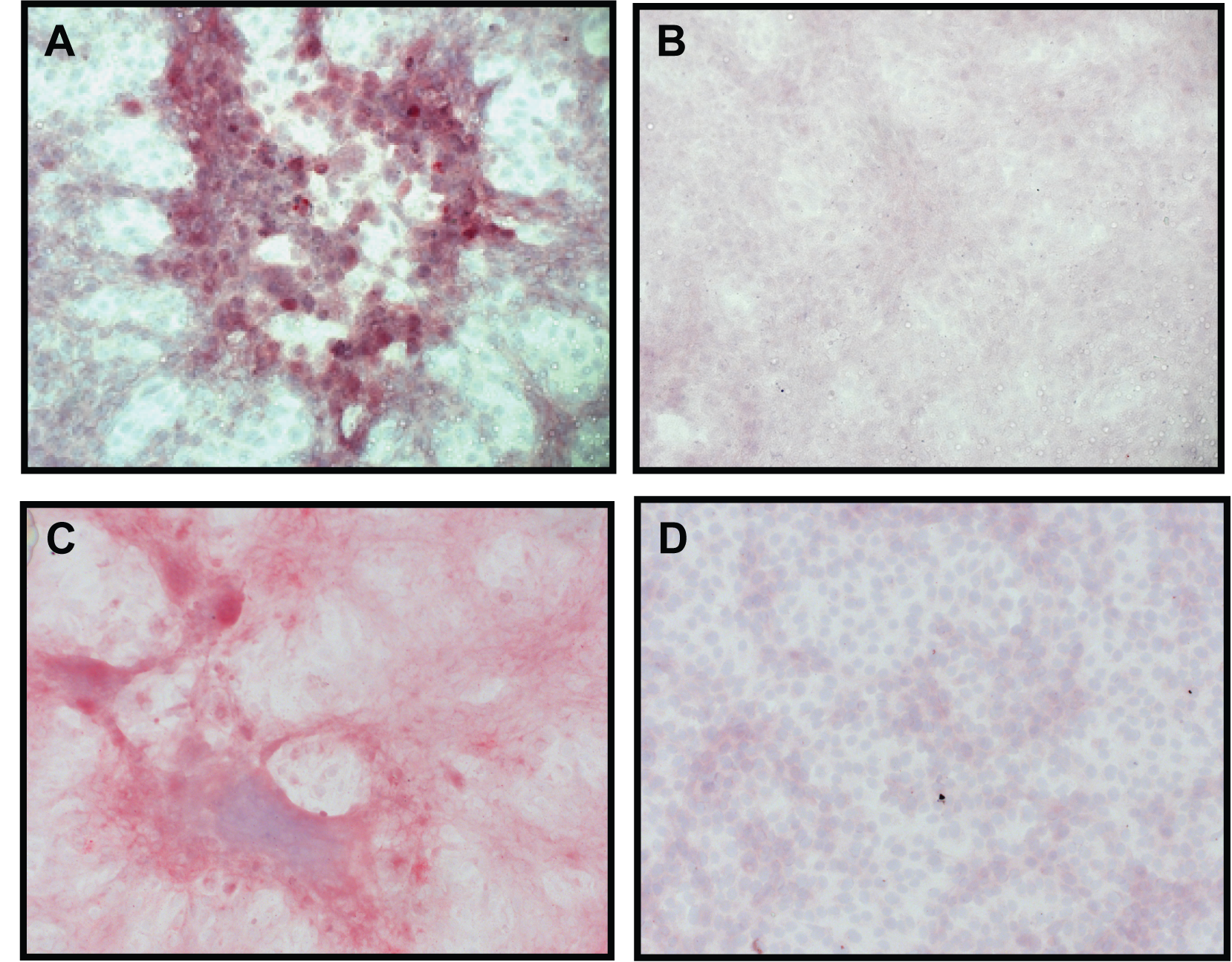

Supplement: Figure S1 — VZV ORF 63- and 61-specific antibodies cross-react with SVV ORF63 and 61 proteins in virus-infected cells. SVV-infected (A, C) and uninfected (B, D) Vero cells in culture were analyzed by immunohistochemistry using a 1∶800 dilution of rabbit anti-VZV ORF 63 (A, B) or a 1∶1000 dilution of rabbit anti-VZV ORF 61 antiserum (C, D). ORF 61 and 63 proteins were detected only in infected cells. (3.49 MB TIF) [file ppat.1000657.s001.tif]
